# Enoxaparin sodium combined with magnesium sulfate in the treatment of severe preeclampsia

**DOI:** 10.12669/pjms.40.5.9001

**Published:** 2024

**Authors:** Dawei Lu, Jie Yu, Lin Sang

**Affiliations:** 1 Dawei Lum, Department of Gynecology and Obstetrics, Hefei Second People’s Hospital, Intersection of Guangde Road and Leshui Road, Hefei City, Anhui Province 230000, P.R. China; 2 Jie Yu, Department of Gynecology and Obstetrics, Hefei Second People’s Hospital, Intersection of Guangde Road and Leshui Road, Hefei City, Anhui Province 230000, P.R. China; 3 Lin Sang, Department of Gynecology and Obstetrics, Hefei Second People’s Hospital, Intersection of Guangde Road and Leshui Road, Hefei City, Anhui Province 230000, P.R. China

**Keywords:** Enoxaparin sodium, Magnesium sulphate, Neonatal Apgar score, Retrospective analysis, Severe preeclampsia

## Abstract

**Objective:**

To observe the treatment of severe preeclampsia in newborns with enoxaparin sodium combined with magnesium sulfate.

**Methods:**

A retrospective analysis was conducted on the clinical data of 80 patients with severe preeclampsia admitted to Hefei Second People’s Hospital, China from January 2019 to December 2020. Treatment records showed that 40 cases received magnesium sulfate treatment (single group), and 40 cases received enoxaparin sodium combined with magnesium sulfate treatment (combination group). Levels of D-dimer, soluble fms-like tyrosine kinase 1 (sFlt-1), *placental growth factor* (PLGF), Apgar scores of newborns delivered before and after treatment were compared. Gestation weeks and incidence of adverse reactions were analyzed.

**Results:**

After treatment, levels of D-dimer, sfit-1 and adverse reactions in the combination group were significantly lower than those in the single group (*P*<0.05), and the level of PLGF, newborn Apgar score and length of gestation were significantly higher than those in the single group (*P*<0.05).

**Conclusion:**

Compared to magnesium sulfate alone, the combination of enoxaparin sodium and magnesium sulfate in the treatment of pregnant women with severe preeclampsia can more effectively regulate the cytokine level of patients, improve pregnancy outcome, and improve neonatal Apgar score. The incidence of adverse reactions is low, making it a safe and efficient treatment modality.

## INTRODUCTION

Preeclampsia is a serious blood pressure condition that develops after the 20th week of pregnancy and is associated with 2% to 8% of pregnancy-related complications worldwide.^1^ Severe preeclampsia is characterized by persistently increase in blood pressure, dizziness, abdominal pain, blurred vision, obvious abnormal changes in liver function and renal function, and impaired lung function and fetal development.^1^,^2^

At present, drug treatment is mainly used for patients with severe preeclampsia.^3^ Magnesium sulfate is a commonly used drug that inhibits central nervous system, relaxes skeletal muscles, and has sedative, antispasmodic, and intracranial pressure-lowering properties.^3^,^4^ However, magnesium sulfate alone may have toxicity and lead to side effects that can seriously affect the vital signs of patients and even pose a life threat.^3^-^5^ Enoxaparin sodium is a low molecular weight heparin preparation that acts as an anticoagulant and prevents thrombosis by acting on the corresponding coagulation factors.^3^ In combination with magnesium sulfate treatment for patients with severe preeclampsia, enoxaparin sodium can treat the hypercoagulable state of pregnant women, help to continue pregnancy or improve the hemodynamic performance during pregnancy, and may play the role in fetal protection.^6^,^7^ However, there are few studies on the effect of enoxaparin sodium combined with magnesium sulfate in the treatment of severe preeclampsia. The main objective of this study was to further clarify the therapeutic effect of the combination of magnesium sulfate and enoxaparin sodium on patients with severe preeclampsia.

## METHODS

Clinical data of 80 patients with severe preeclampsia treated in Hefei Second People’s Hospital from January 2019 to December 2020 were analyzed retrospectively. The patients were divided into two groups according to the treatment scheme they received: single group (received magnesium sulfate alone, n=40) and the combination group (received magnesium sulfate combined with enoxaparin sodium, n=40).

### Ethical Approval:

The ethics committee of our hospital was fully informed of the study and approved the study (Approval No.: 2018121801; Date: 12-December, 2018). All patients and their immediate family members voluntarily participated in the study after knowing the contents of the study in detail and signed relevant agreements.

### Inclusion criteria:

- Patients met the American College of Obstetricians and Gynecologists (ACOG)’s diagnostic criteria of severe preeclampsia.^8^
- Singleton live births.
- Complete medical data.

### Exclusion criteria:

- Patients with severe organic diseases, primary hypertension, diabetes, coagulopathy and psychiatric history.
- Patients with language, cognitive, or mental disorders.
- Allergic to the drugs used in this study.
- Those who were about to give birth or underwent surgery.
- Premature rupture of membranes.
- Severe myocardial damage.

### Therapeutic method:

All patients received routine treatment, including conventional antispasmodic, antihypertensive, and sedation. During the treatment, patients were closely monitored for changes in heart rate and blood pressure, as well as changes in fetal heart rate, and were guided to adopt a high-protein, low-fat diet.

Patients in the single group were given magnesium sulfate injection (Yangzhou Zhongbao Pharmaceutical Co., Ltd; National approval number H32024805). Administration: First, 2.5g magnesium sulfate injection was diluted into 150ml mixture by adding 25% glucose injection, and intravenous infusion was completed within 30min. Then 7.5g magnesium sulfate injection was mixed with 25% glucose injection to 600ml for intravenous infusion, and the administration rate was 1.5-2.0g /h. The maximum daily dosage of magnesium sulfate should be less than 25g, and the treatment course was seven days.

In the combination group, enoxaparin sodium injection (Hangzhou Jiuyuan Genetic Engineering Co., Ltd; National approval number H20064067) was added on the basis of the treatment of a single group. Administration: Enoxaparin sodium injection was injected subcutaneously, 0.3 mL/time, once/day, and continued for seven days.

### Observation index:

Levels of cytokines (D-dimer, placental growth factor [PlGF], human soluble FMS like tyrosine kinase-1 [sfit-1]), Apgar score of newborns, gestational weeks and the incidence of adverse reactions (palpitation and sudden drop of blood pressure) were compared and analyzed before and after treatment.

### Statistical analysis:

SPSS 20.0 was used for data analysis. Normally distributed measurement data was reported as mean and standard deviation (SD), and compared using student t-test. Non-normally distributed data was reported as medians and interquartile intervals, and compared using Mann Whitney U test. Counting data were reported as n (%), and compared using Chi-squared test. *P*<0.05 was considered statistically significant.

## RESULTS

A total of 80 patients were included in this study. The single group included 16 primiparas and 24 multipara women, and the age of the patients ranged from 22 to 36 years with a mean of 29.95±2.92 years. The combination group included 15 primiparas and 25 multipara women, and the age of the patients ranged from 24 to 35 years with a mean of 29.03±2.87 years. There was no significant difference in the general characteristics (*P*>0.05) (Table-I).

**Table-I:** Comparison of baseline characteristics between the two groups

Group	n	Age (year)	primiparas/ multipara (n)	pre-pregnancy BMI (kg/m^2^)
Single group	40	29.95±2.92	16/24	24.84±3.97
Combination group	40	29.03±2.87	15/25	24.82±4.10
*t*/ *χ*² continuity correction	-	1.430	0.053	0.025
*P*	-	0.157	0.818	0.980

There was no significant difference in cytokine levels between the two groups before treatment (*P*>0.05. After treatment, D-dimer and sfit-1 in the combination group were significantly lower than those in the single group (*P*<0.05), and PLGF levels were significantly higher than those in the single group (*P*<0.05) (Table-II). The Apgar scores of newborns delivered by the patients in the combined group at different times were significantly higher than those in the single group (*P*<0.05) (Table-III). Length of gestation (weeks) was significantly higher, and the incidence of adverse reactions was significantly lower in the combined group than in the single group (*P*<0.05) (Table-IV).

**Table-II:** Comparison of cytokine levels between the two groups before and after treatment.

Status	Group	D-Dimer (μg·L^-1^)	PLGF (ng·L^-1^)	sFIt-1 (ng·L^-1^)
Before treatment	Single group (n = 40)	562.25±62.98	40.55±4.61	1576.65±162.80
	Combination group (n = 40)	557.43±68.30	41.00±4.84	1547.40±176.45
*t*	-	0.328	-0.426	0.771
*P*	-	0.743	0.671	0.443
After treatment	Single group (n = 40)	367.43±49.36*	48.83±7.13*	1427.38±130.00*
	Combination group (n = 40)	272.28±52.05*	58.18±6.51*	1074.38±140.54*
*t*	-	8.390	-6.125	11.662
*P*	-	0.000	0.000	0.000

***Note:***

* indicates that compared with the same group before treatment, *P*<0.05.

**Table-III:** Comparison of Apgar scores of newborns delivered between the two groups.

Group	n	Apgar score at one minute after birth	Apgar score at five minute after birth
Single group	40	8 (7,9)	8 (7,10)
Combination group	40	9 (8,10)	10 (9,10)
*Z*	-	-3.180	-4.007
*P*	-	0.001	0.000

**Table-IV:** Comparison of gestational weeks and incidence of adverse reactions between the two groups.

Group	n	Gestational week (weeks)	Palpitation	Sudden drop in blood pressure	Incidence of adverse reactions (%)
Single group	40	35 (34,36)	2 (5.00)	6 (15.00)	8 (20.00)
Combination group	40	38 (38,39)	0 (0.00)	1 (2.50)	1 (2.50)
*Z*/ *χ*² continuity correction	-	-6.341	-	-	4.507
*P*	-	0.000	-	-	0.034

## DISCUSSION

The results of this study show that compared to magnesium sulfate alone, the combination of enoxaparin sodium and magnesium sulfate in the treatment of severe preeclampsia can more effectively regulate patient’s cytokine levels, improve neonatal Apgar scores, and have a lower incidence of adverse reactions.

Wen J et al.^9^ showed that the efficacy of low molecular weight heparin sodium combined with magnesium sulfate in the treatment of severe preeclampsia patients is significantly higher than that of patients treated solely with magnesium sulfate. Wang N et al.^10^ also showed that low-dose low-molecular-weight heparin combined with magnesium sulfate can promote the expression of endogenous kallikrein, weaken the infiltration of the cytotrophoblast, and improve the maternal coagulation state in severe PE patients. It can also improve placental microvascular flow and effectively prolong pregnancy, promote fetal growth, and improve clinical outcomes.^10^ The results of this study are consistent with the above research. The combination of enoxaparin sodium and magnesium sulfate in the treatment of severe preeclampsia can inhibit hypercoagulation, prevent spasms, lower blood pressure, and is the key to improving clinical symptoms in patients.^9^,^10^,^11^

At present, patients with severe preeclampsia are mainly treated with antispasmodic and sedative drugs to slow down the progression of the condition and prolong the gestation.^3^,^8^ Magnesium sulfate, as the main antihypertensive drug for acute hypertension, is the first choice for patients with severe preeclampsia, mainly because magnesium sulfate is able to reduce blood pressure and relieve muscle convulsion by inhibiting the activity of the central nervous system, blocking conduction at the neuromuscular junctions, reducing or relieving muscle contractions, relaxing vascular smooth muscles, and dilating the spasmodic peripheral blood vessels.^5^,^12^ However, the antihypertensive effect of the single use of this drug is relatively slow, and may lead to the withdrawal symptoms in patients. Therefore, the key to the effective treatment of severe preeclampsia with magnesium sulfate is to use drug combinations to improve treatment efficiency and drug safety.^13^,^14^

Enoxaparin sodium, a low molecular fragment extracted from unfractionated heparin, is a vitamin K antagonist with high anticoagulant effect and has been commonly used in clinical practice. As a low molecular weight heparin preparation, enoxaparin sodium has strong antithrombotic effect and certain thrombolytic effect.^15^ In combination with magnesium sulfate treatment in patients with severe preeclampsia, antithrombin III and its complex can enhance the inhibitory effect of antithrombin III on activated coagulation factors II, IX, X, Xi and XII to prevent the transformation of fibrinogen into fibrin, prevent platelet aggregation, destroy and hinder thrombin activity, thus preventing blood hypercoagulability.^16^,^17^ D-dimer is a specific degradation product formed after plasmin hydrolyzes cross-linked fibrin. It is one of the molecular markers of hypercoagulable state and secondary hyperfibrinolysis in vivo. As a degradation product of cross-linked fibrin, it is a unique metabolite of secondary fibrinolysis and an important indicator of fibrinolytic function. Placental growth factor (PLGF) is a highly specific marker secreted by the placenta that regulates endothelial cell function and promotes neovascularization, and is considered an important indicator reflecting placental function. The decrease in PLGF level often indicates placental insufficiency, and patients are prone to complications such as miscarriage and preeclampsia. Soluble fms-like tyrosine kinase 1 (sFlt-1) is an important index reflecting the growth of placental blood vessels. The increase in its level can lead to endothelial dysfunction, and subsequently affect the shaping process of placental spiral arteries, resulting in abnormal placental perfusion.^18^ Both sFlt-1 and PLGF are considered early biomarkers of placental function, and the changes in their concentrations significantly precede the onset of preeclampsia.^19^

This study aimed to further clarify the clinical value of enoxaparin sodium combined with magnesium sulfate in the treatment of pregnant women with severe preeclampsia. Our results indicate that the incidence of elevated levels of D-dimer, sFlt-1 and adverse reactions in the combined group were significantly lower than those in the single group treated with magnesium sulfate alone. Patients in the combined group had higher levels of PLGF. Combination of magnesium sulfate with enoxaparin sodium was associated with longer gestational period and higher Apgar scores (at different time points) of newborns as compared to patients treated with magnesium sulfate alone. Our results indicate that enoxaparin sodium combined with magnesium sulfate is safe and effective in the treatment of severe preeclampsia, and can effectively adjust the levels of D-dimer, PLGF and sFlt-1, prolong the gestation period, prevent neonatal asphyxia, and improve physical condition of newborns.^9^,^10^,^18^ We speculate this effect of enoxaparin sodium may be due to its ability to promote the release of endogenous heparin, maintain the dynamic balance of coagulation and fibrinolysis system, and strengthen the antithrombotic effect.^20^ Moreover, since enoxaparin sodium can decrease vasoconstriction, it may improve hypercoagulable state of pregnant women, as well as the hemodynamic performance, blood perfusion and placental circulation, and alleviate the oxygen supply pressure of placental syncytiotrophoblast cells, thus positively affecting physical condition of newborns.^21^

Our results suggest that enoxaparin sodium combined with magnesium sulfate can produce a synergistic effect, which can effectively improve the fibrinolytic activity, reduce the release of neurotransmitters, reduce blood flow resistance, improve local microcirculation, reduce organ ischemia and hypoxia, improve fetal blood supply and placental hemodynamics, and optimize maternal and infant outcomes.^22^

### Limitations:

The current study is retrospective and has a small sample size, which may be biased and its conclusions should be interpreted with caution. In addition, only a few indicators were studied, others such as 24-hour urinary protein and p-selectin levels were not studied. Furthermore, longer follow-up is needed to investigate the long-term health status and potential recurrence rate of patients.

## CONCLUSION

Compared to magnesium sulfate alone, the combination of enoxaparin sodium and magnesium sulfate in the treatment of pregnant women with severe preeclampsia can effectively adjust the cytokine level of patients, improve pregnancy outcome, and improve neonatal Apgar score, with a low incidence of adverse reactions. It is a safe and efficient treatment modality.

### Authors’ contributions:

**DL:** Conceived and designed the study.

**JY** and **LS:** Collected the data and performed the analysis.

**DL:** Was involved in the writing of the manuscript and is responsible for the integrity of the study.

All authors have read and approved the final manuscript.
